# Where, why and who delivers our babies? Examining the perspectives of women on utilization of antenatal and delivery services in a developing country

**DOI:** 10.1186/s12884-022-05306-6

**Published:** 2023-01-02

**Authors:** Edmund Ndudi Ossai, Irene Ifeyinwa Eze, Pearl Chizobam Eke, Cosmas Kenan Onah, Chibuike Agu, Lawrence Ulu Ogbonnaya

**Affiliations:** 1 Department of Community Medicine, College of Health Sciences, Ebonyi State University Abakaliki, Abakaliki, Nigeria; 2 Department of Community Medicine, Alex-Ekwueme Federal University Teaching Hospital Abakaliki, Abakaliki, Nigeria; 3 Department of Nursing Services, Alex-Ekwueme Federal University Teaching Hospital Abakaliki, Abakaliki, Nigeria

**Keywords:** Antenatal care, Delivery, Utilization, Urban, Rural Ebonyi state, Nigeria

## Abstract

**Background:**

The differences in maternal mortality between developed and developing countries is due to differences in use of antenatal and delivery services. The study was designed to determine the views of women on utilization of antenatal and delivery services in urban and rural communities of Ebonyi state, Nigeria.

**Methods:**

Community based descriptive exploratory study design was employed. Qualitative data was collected through use of pre-tested focus group discussion (FGD) guide. Eight FGDs were conducted among women who were pregnant and others who have delivered babies one year prior to the study. Four FGDs each were conducted in urban and rural communities. QDA Miner Lite v2.0.6 was used in the analysis of the data.

**Results:**

Most of the participants in urban and rural areas prefer the man and woman deciding on where to receive antenatal and deliver care. All the participants in urban and rural communities wish for the support of their husbands when pregnant. Perceived quality of care is the major reason the women choose a facility for antenatal and delivery services. Others reasons included cost of services and proximity to a facility. Participants in rural communities were of the opinion that traditional birth attendants deliver unique services including helping women to achieve conception. For participants in urban, traditional birth attendants are very friendly and perhaps on divine assignment. These reasons explain why women still patronize their services. The major criticism of services of traditional birth attendants is their inability to manage complications associated with pregnancy and delivery. The major reasons why women delivery at home included poverty and cultural beliefs.

**Conclusions:**

All efforts should be made to reduce the huge maternal death burden in Nigeria. This may necessitate the involvement of men and by extension communities in antenatal and delivery matters. There is need to train health workers in orthodox health facilities on delivery of quality healthcare. Public enlightenment on importance of health facility delivery will be of essence. Encouraging women to deliver in health facilities should be prioritized. This may entail the provision of free or subsidized delivery services. The deficiencies of primary health centers especially in rural communities should be addressed.

## Background

Maternal mortality more than any other health indicator reveals the greatest disparity between the high income and low income countries of the world [1]. For instance while the life time risk of maternal death in sub-Saharan Africa is very high and estimated at 1 in 37 that for Australia and New Zealand is estimated at 1 in 7800 [2]. Expectedly, sub-Saharan Africa accounted for 66% of the estimated 295,000 maternal deaths in the world in the year 2017 [2]. Furthermore, eighteen of the nineteen countries with the highest maternal mortality ratios in the world are all in the region of Africa [2]. In these countries with very high maternal mortality ratios, there is evidence that women are less likely to be attended to by a skilled health worker during delivery [3]. It is of importance to note that only three countries in sub-Sahara African region including Mauritius, Cape Verde and Seychelles have low maternal mortality ratios of between 53 and 61 maternal deaths per 100,000 live births [2].

On the country level, Nigeria accounts for 23% of global maternal deaths and thus bears the highest burden of maternal deaths globally [2]. The maternal mortality ratio in Nigeria is estimated at 512 maternal deaths per 100,000 live births and a lifetime risk of maternal death of one in 34 women [4]. In Nigeria, there are variations in the utilization of maternal health services. For example, while 67% of pregnant women received antenatal care from a skilled provider, 39% of deliveries took place in a health facility [4[. Also, women living in the urban areas are more likely to receive antenatal and delivery care from a skilled provider when compared with those that live in rural communities [4]. Similarly, women who make four or more antenatal care visits are more likely to deliver in a health facility when compared with those who made no antenatal care visits [4].

Also, there is a positive relationship between use of antenatal care and being attended to by a skilled attendant during delivery [5]. This is because antenatal care visits may provide opportunities for health education including the benefits of utilizing a skilled birth attendant at delivery. Suffice it to say that skilled attendant at delivery has been identified as the most effective strategy in reducing maternal and neonatal morbidity and mortality [6, 7]. Thus, the differences as observed in maternal mortality ratio between the developed and developing countries could be due to the differences in use of antenatal and delivery services. On the global scene, one of the targets of Sustainable Development Goal 3 is to reduce the global maternal mortality ratio to less than 70 per 100,000 live births by the year 2030 [8]. Thus in achieving this goal, the use of antenatal care and having a skilled attendant at every delivery is crucial. Unfortunately, the use of health facilities for delivery services in Nigeria is very poor [9–11] and this may be the same in other countries of sub-Saharan Africa.

In a study in southeast Nigeria, providers of maternal health services in urban and rural communities identified ignorance on the values of antenatal care and delivery with a skilled provider as the main obstacle to the use of health facilities for such services [12]. Another study in north-central Nigeria concluded that even community members did not have knowledge of the benefits of health facility delivery [13]. This situation is a bit different from what was found in a study in rural Tanzania where women were aware of the benefits of antenatal and delivery services but were ignorant of the role of medical services at those important periods [14]. In a study in south-south Nigeria, majority of pregnant women perceived antenatal care as being curative instead of preventive. In effect, majority of the women booked late for antenatal care due to misconceptions of the purpose and right time to initiate care during pregnancy [15]. A similar study in rural Kenya revealed that women likened antenatal care as a place for treatment of common diseases [16]. A study in southwest Nigeria however had a different finding. The results revealed that majority of pregnant women had positive perception of the activities and services of traditional birth attendants hence majority were satisfied with the services received from them. Incidentally, majority of the women opposed the banning of the services of traditional birth attendants [17]. These findings have huge implications on efforts towards improving maternal health in Nigeria and other less developed countries of the world.

There is evidence that investments in improving the health of mothers and their babies procure economic benefits to countries [18]. This is because maternal and newborn deaths reduce global productivity by an approximate 15 billion dollars every year [19]. Thus it has been estimated that about one third and one half of Asia’s economic growth from 1965 to 1990 was due to reductions in infant and child mortality and fertility rates [18]. This tallies with the vision of Sustainable Development Goal 3 [8]. It is important to note that in the realization of this goal, there is the need to improve the utilization of antenatal and delivery services by the women. This is of relevance to Nigeria bearing in mind the high maternal death burden in the country. Furthermore, it has been posited that in improving maternal health in Nigeria, the delivery of client oriented services is essential [9] and there is the need to pay attention to what is happening in the rural areas of the country [9] where in the year 2020, close to half of the Nigerian population reside [20] and the maternal death burden is higher [4]. There is evidence that in Nigeria the utilization of skilled providers for maternal health services is highest in southeast geo-political zone when compared with others zones of the country. However, it is on record that inhabitants of Ebonyi state has the least utilization of such services in the zone [4]. This necessitates the need for action towards improving maternal health. This study was designed to determine the views of women on the utilization of antenatal and delivery services in urban and rural communities of Ebonyi State, southeast Nigeria.

## Methods

### Study setting

The study took place in Ebonyi state which is one of the five states in southeast geo-political zone of Nigeria. The inhabitants of the state are mainly of Igbo ethnic nationality. The state is made up of thirteen local government areas (LGAs) of which three are designated as urban which ten are classified as rural. Ebonyi state just like other states in Nigeria operate three levels of healthcare delivery. The primary level is managed by the local government councils, the secondary by the State Government while the Federal Government of Nigeria is in charge of tertiary healthcare. The State has a total of 545 health facilities; 530 are primary healthcare facilities, 13 are secondary health facilities while there are two tertiary health institutions in the State. Among the five states in the southeast geo-political zones, Ebonyi state has the least utilization of antenatal and delivery services with a skilled provider and invariably the worst maternal health index in the zone [4].

### Study design, participants and sampling

This was a community based, qualitative descriptive study. Sixty nine women participated in eight focus group discussions (FGDs). Thirty three of the women were pregnant during the period of study and participated in four focus group discussions. Also, thirty six women who delivered their babies at least one year before the commencement of the study participated in four focus group discussions. Four of the FGDs took place among the women who reside in urban areas of the state while the remaining four FGDs took place among the inhabitants of rural communities in the state.

A two stage sampling process was used in the recruitment of participants for the study. In the first stage, a simple random sampling technique of balloting was used to select two LGAs from the three urban LGAs and another two from ten LGAs designated as rural. In the second stage, two communities were selected from a list of all communities in each of the selected LGAs using a simple random sampling technique of balloting. The communities are as defined by the Government of Ebonyi State, Nigeria, being a group of people that share the same values and have a single leadership structure usually headed by a traditional king. The participants in the study were purposively selected from the selected communities with the help of community guides. One FGD took place in each of the eight selected communities.

### Study instrument and data collection method

Information was obtained from the participants using a pre-tested focus group discussion guide. The pre-testing was done in a community in a LGA of the state not selected for the study. The aim of the pre-testing was to identify and correct ambiguities of the study instrument. The FGDs were conducted using English language depending on the participant and the discussions took place in public places like primary schools in the community. All the discussions were recorded manually with the aid of a note taker and with a digital recorder. Personal contacts were made with all the participants with the help of the community guides after which a date was fixed for each of the discussions.

The note taker summarized the responses of the participants in detailed notes. Follow up questions using probes were asked during the interviews as a way of having good understanding of the subject at hand if the explanation was unclear. The average duration of the discussions was 58 min.

### Data management

The data management process has been described in a previous publication [12]. Qualitative data analysis (QDA) Miner Lite v2.0.6 was used in the analysis. Six themes emerged from the FGDs and included who takes decisions on where a woman attends antenatal and delivery care, places where women go for antenatal and delivery services and use of traditional birth attendants for antenatal and delivery care. Others included opinions of women concerning traditional birth attendants, reasons why women deliver at home and role of men in issues related to antenatal and delivery care.

## Results

### Interviewer characteristics

This has been described in a previous publication [12].

### Participants’ profile

The age range of study participants in the urban area was 24 to 35 years and the median age was 31 years. In the rural area, it was 20 to 34 years with a median age of 26 years. Most of the participants in the urban area, 70% have attained tertiary education while in the rural area, 60% of the participants have had secondary education. Most of the participants in the urban area were on salaried employment while in the rural area most were self-employed.

### Decisions concerning where a woman attends antenatal and delivery care

Most of the discussants in the urban and rural areas were of the opinion that the decision on where a woman should obtains antenatal and delivery care should be a joint decision between the man and the woman. They were of the opinion that this approach will make the decision very firm. A participant in the urban area presented her views succinctly:

*“This is a family matter and as such the husband and wife should be in agreement on where the woman should attend antenatal care and also deliver her baby”* (Discussant, urban area)

Even though most of the participants agreed that it should be a decision made by the man and the woman, among the remaining respondents, some of them were eager to give either party an upper hand in the decision making process. In this regard, most of the participants in the rural area preferred the women having an edge in deciding where a woman should receive antenatal and deliver care. This is how a discussant in the rural area expressed her thoughts:

*“It is the woman that should have the final say on where to attend antenatal care and also delivery her baby because it is for her own good since she is the one that is pregnant. In-fact all matters related to safe delivery are centered on the woman and as such she should be more involved in all decisions in such matters”* (Discussant, rural area)

Some of the participants in the urban pointed to the very important role of men in matters related to antenatal and delivery which may eventually give them the edge in the making of decisions. This was how one of the participants expressed her thoughts:

*“Husbands are important in giving direction, it is their responsibility to give orders especially when things are no longer straight forward like when there is an emergency”* (Discussant, urban area)

Another participant related her experience in a way as to portray that the man should have the final say in such matters. This was her narration:

*“Husbands have the power to ensure that their wives go for antenatal care. For instance in my first pregnancy, my husband insisted I must go to hospital. I was aware that my mother delivered all her six children including me at home without going to hospital and so was willing to stay at home provided I am not sick till the day I will deliver my baby but my husband insisted I must go to hospital for antenatal care and because of that I registered for antenatal care”* (Discussant, urban)

A few of the participants who were in support of a joint decision offered explanations on when it becomes necessary that the woman should be the one to take charge of her health. These were captured in the following two quotes:

*“It is the woman and her husband that should decide on when and where the woman should attend antenatal care and deliver the baby but if the husband is one of those men who show ‘I don’t care attitude’ then the woman should take care of herself and if she has the money pay the hospital bills also”* (Discussant, rural)

*“Some men are stingy and will want their wives to go for antenatal care in places that will cost less or no money. Under such conditions, the woman should be able to make the decision on where she should attend antenatal care and also deliver the baby”* (Discussant, urban)

One of the participants in the urban area also pointed out other groups of people who also decide on where women attend antenatal care and also deliver. She named the mother of the woman, mother-in-law and other women in the neighborhood especially those who have experience in matters related to childbirth.

### Role of men in antenatal and delivery care

All the participants in the urban and rural communities want intimacy with their husbands whenever they are pregnant. They were of the opinion that the provision of all material and financial needs during the period of pregnancy by the husband should be a ‘settled matter’. The main focus of this intimacy was to ensure they are happy all through the pregnancy period. The thoughts of the women are explained in the following quotes:

*“Husbands should always try to make their pregnant wives happy, understand their moods, feel their feelings, show concern and endeavour to reassure the women that all will be well”* (Discussant, urban)

*“The role of our husbands when we are pregnant is more than financial. They should not provoke us but always try to make us happy, encourage us and provide all forms of support needed for a healthy mother and baby”* (Discussant, rural)

A participant in the urban area went further to explain the benefits of intimacy among couples during the pregnancy period. This was how she explained it:

*“Physical touch and intimacy of the husband with the wife during pregnancy will ensure that the baby in the womb will share both parents’ blood instead of that of the mother alone if intimacy was absent”* (Discussant, urban)

Even though all the participants in the urban and rural communities wanted their husbands to remind and encourage them to attend antenatal care, the two groups of women differed on the subject of husbands accompanying them for antenatal care and during delivery. Most of the participants in the urban area were in favour of husbands accompanying their wives to antenatal care and also be around when they are to deliver. One of the participants had this to say:

*“Our husbands should accompany us during antenatal care visits. They should be around us when it is time for delivery, assisting in everything that we may need. In-fact they must not travel when our date of delivery is due”* (Discussant, urban)

The reverse was the case for participants in the rural area as almost all of them did not approve of the man accompanying them for antenatal care visits. One of the participants presented her views this way which was chorused yes by all the participants:

*“Our husbands should not accompany us for antenatal care, the result of such a mission will be a reduction in the money they give us when we go for antenatal care. We (the pregnant women) will attend our antenatal care ourselves”* (Discussant, rural)

The participants in the rural area however had no objections to their husbands accompanying them or being around during hospital visits when the purpose is to deliver.

### Places women go for antenatal care and delivery services

Most of the discussants in the urban and rural areas indicated that they attend antenatal care and also deliver their babies at private and government hospitals including primary health centers. However some of the women also patronize maternity homes and traditional birth attendants. The women also pointed out what is relatively unknown that a few women make use of prayer houses and patent medicine vendors for antenatal and delivery care. It was also learnt that some women deliver at home assisted by a birth attendant or any other woman who may be around during the time of delivery.

### What determines where a woman should attend antenatal care and obtain delivery services?

Most of the participants in the urban and rural areas were of the opinion that perceived quality of care in a health facility is a major factor the woman considers in deciding on where to register for antenatal care and deliver her baby. This perceived quality of care could be based on the experiences of the woman or following the opinion of other women and cuts across the different classes of health facilities. The experiences of the women were exemplified by the following quotes:

*“I prefer to go to a hospital but not just ‘anyhow hospital’ but a hospital with good reputation of taking good care of women and with specialist doctors in the field of Obstetrics and Gynecology who are well trained to manage labour very well”* (Discussant, urban)

*“The quality of care, in that health facility is important, for instance in my first and second deliveries in a private hospital, the doctors and nurses were very caring, polite and very concerned. They never shouted at me unlike what is seen in the government hospitals. As such I have decided to deliver all my babies there”* (Discussant, urban)

It was also found that the community members have a role to play in defining the concept of good quality care and sometimes this may not be based on the concept of a skilled provider. One of the participants had this to say:

*“What the community or the women say about a health facility makes us (the pregnant women) to decide whether to deliver there or not. The health workers in some health centers are seen or reputed to be caring and such a report supports delivery in such a facility. In-fact that is why the traditional birth attendants are thriving because our mothers, mothers-in-law and neighbours who have patronized them before spread good news about them in the community and that explains why they are still patronized for antenatal and delivery care in the community till this day”* (Discussant, rural)

For a few of the participants, previous experience in a health facility helps in categorizing a particular facility as being of good standard.

*“Your previous experience in a health facility has a role to play. In my first pregnancy, I went to antenatal care in a health facility and the doctor did not care. He permitted someone that does not ‘know anything about my pregnancy’ to do vaginal examination and that led to premature rupture of membrane that eventually led to the death of one of my twins. Because of this, I will never go to that health facility or advise anyone to go there”* (Discussant, urban)

The cost of delivery is another factor that influences where the woman registers for antenatal care or plan for delivery in the urban and rural areas. One of the participants in the urban area had this to say:

*“You know that government and private hospitals do not charge same fees for antenatal and delivery services. So the financial strength of the woman determines whether she will attend antenatal care and deliver in a government or private hospital”* (Discussant, urban)

Among the participants in urban and rural areas, closely following cost of services is the proximity of the health facility to the home of the woman. A participant in rural area speaking specifically for delivery services had this to say:

*“How close a health facility is to one’s home is very important. I prefer a facility that is close to my home so that anytime labour starts, I don’t need to start arranging for transport, I simply walk to the health facility”* (Discussant, rural)

### Use of traditional birth attendants for antenatal and delivery care

The participants gave several reasons people still patronize the traditional birth attendants for antenatal and delivery services. However, participants from the rural area emphasized the unique services of traditional birth attendants. A participant who inferred that traditional birth attendants help women achieve conception made this remark:

*“In situations where the traditional birth attendant assisted the woman in getting pregnant especially those who had difficulty in conceiving, then they are most likely to attend antenatal care with the traditional birth attendant and also deliver there”* (Discussant, rural)

Another participant from the rural area also gave indication that the traditional birth attendants also provide other services apart from antenatal and delivery services which is a plus for their clients. This was how one of the participants made her views known:

*“You see, there are some disease conditions associated with pregnancy that requires herbal drugs for complete cure such as “iba’ (referring to malaria) and ‘okpe’ (meaning helminthiasis). Pregnant women who patronize traditional birth attendants are given herbal medicines which help them pass out these diseases in the urine”* (Discussant, rural)

Another participant also in the rural area collaborated to this by indicating that the herbal medicines from the traditional birth attendants also cure a form of ‘internal heat’ mostly felt at the pelvic region during pregnancy. She went further to indicate that the herbal medicines have a positive effect on a contracted pelvis. Her view was expressed this way:

*“The herbal medicines from the traditional birth attendants when taken by women with contracted pelvis will enable free movement of the pelvic bones during labour thus enabling the women to deliver on their own without an operation”* (Discussant, rural)

The participants in the urban area had a different impression of why people patronize the traditional birth attendants. The most important being the good services they provide. This was how a participant made it known:

*“The traditional birth attendants know how to deliver good healthcare, they deliver their services in a very friendly manner unlike the public health facilities where the workers are careless and very less concerned in the way they deliver services”* (Discussant, urban)

There is also a feeling among the people that the traditional birth attendants are on divine assignment. This was how a participant in the urban expressed her views:

*“Some women believe that the traditional birth attendants are naturally gifted to deliver new born babies and that this gift is from God and when people recall that their mothers delivered there safely and women still deliver there safely till today they are convinced that it is the best place to deliver”* (Discussant, urban)

The participants in the urban and rural areas also noted that ignorance and lack of money coupled with the high medical bills charged by both public and private health institutions play very key roles on why the women still patronize traditional birth attendants. One participant in the rural area remarked that lack of access roads to health facilities in the rural area also encourage the women to patronize traditional birth attendants.

### The opinion of the women of traditional birth attendants

The participants’ views of the services of traditional birth attendants included a criticism of their services. This criticism centered on their inability to manage emergency situations which the participants were aware could occur in pregnancy. This observation was the same among participants in urban and rural communities. A participant in the urban area was quick to focus on issues related to the placenta. She presented her views this way:

*“It is not good to patronize traditional birth attendants for delivery services because in case of an emergency, e.g. placenta previa or any other complication they cannot be of assistance and before one could seek help in another place, the woman may be in critical condition or even die”* (Discussant, urban)

Perhaps some of the participants were aware of the dangers of bleeding post-delivery and saw this as a deterrent to patronizing the traditional birth attendants for delivery services. These were summarized in the following two quotes:

*“There is a problem in going to traditional birth attendants to deliver your baby because in cases of severe bleeding that may necessitate blood transfusion, the traditional birth attendant may not be able to stop the bleeding and cannot transfuse blood also. That means that under such situations, they can do nothing and that is dangerous”* (Discussant, urban)

*“Even though the traditional birth attendants are good in delivering women of their children, in cases where there is bleeding or convulsion, they cannot really help the woman and that is why going to hospital where there are qualified health workers is better”* (Discussant, rural)

One participant also supported this assertion based on her previous experience in labour and emphasized the importance of trained health workers. She shared her experience this way:

*“It is good to go to hospital because sometimes complications could arise, for instance in the delivery of my last baby, at a certain point the baby was stock at the birth canal and it took the intervention of the doctors using a ‘force machine’ (meaning a vacuum extractor) to assist me and deliver my baby. That is why I say that trained health workers make the difference, they are better than traditional birth attendants”* (Discussant, urban)

One of the participants in the rural area however rose to the defense of the traditional birth attendants emphasizing that they have several remedial measures for some emergencies that may arise during the delivery process. Two participants in urban and four from the rural area were particular about the unhygienic practices of the traditional birth attendants. One participant from the urban area remarked that the traditional birth attendants focus more on the woman with little or no skills for the management of the newborn baby. According to her, they have no equipment to examine the newborn baby and cannot resuscitate the baby if the need for that arises. Another participant in the urban area was spiritual in her approach to the activities of the traditional birth attendants. She presented her views this way:

*“Some of the traditional birth attendants may be operating with a ‘bad spirit’ which they can pass on to the child during the delivery process and this can affect the child adversely”* (Discussant, urban)

### Reasons why women deliver at home

Most of the participants in both the urban and rural areas identified lack of finance as the major reason why women deliver their babies at home. Most of the participants were of the opinion also that the hospital bills in both government and private hospitals are too high thus worsening the financial situation. These were how the women expressed their views:

*“Lack of money and most times, no means of even going to the hospital make people to deliver at home. Some women instead of suffering themselves and their families will prefer to leave everything in the hand of God hence deliver their babies at home”* (Discussant, urban)

*“Most of the women who deliver at home do so because of financial problems. You see the economy is not good and some of the hospitals charge very high bills for delivery which they cannot afford and because of this many will just deliver at home”* (Discussant, rural)

A close scrutiny may reveal that this concept of no money is individually defined and may be due to low priority placed on delivery. A participant had this to say about a friend and home delivery:

*“Some people deliver at home mainly due to lack of money. My colleague though she works in a hospital delivered all her children at home due to the high bills charged for delivery in our hospitals”* (Discussant, urban)

Perhaps, this matter of money limiting access to delivery services in hospitals prompted one of the participants to plead with the government to subsidize hospital delivery fees or make it completely free. She had this to say:

*“I think that the government should subsidize antenatal and delivery services or make it free so that those running away from our hospitals due to financial problems can easily access care”* (Discussion, urban)

Another participant in the urban area viewed all these excuses around money limiting access to antenatal and delivery services as due to ignorance. She remarked about her friend this way:

*“My friend claimed that her mother delivered all of them (seven) at home, and being her child, she inherited that characteristic and as such she has no need for antenatal care and delivery in a hospital. So she delivers her children at home”* (Discussant, urban)

Another major reason for delivering at home was related to culture. It is perceived that a true woman does not need the assistance of anyone to deliver a baby. One of the participants likened this to the Fulani women who due to their culture can bear pain and hence deliver easily at home. They however pointed out this is more prevalent in the rural areas. This was how this information was collaborated by participants in urban and rural area:

*“Some said that delivery at home shows strength hence any woman who delivers her baby at home is seen as a strong woman and will be praised by the people especially the elderly women in the community”* (Discussant, rural)

*“In some villages, they view the women going to deliver in hospitals as being weak and that if anyone goes to a hospital to deliver, the doctors and nurses will install fear in the person. Child birth is a proof of womanhood hence a true woman delivers at home”* (Discussant, urban)

Closely related to the cultural influence in limiting access to delivery services is the demystification of pregnancy and delivery by women after passing through the experience. In this regard, the women now view pregnancy as normal issue they could handle on their own. One of the participants presented her thoughts this way:

*“Some women who have delivered more than two children feel that it is no longer useful going to the hospital to deliver since they are now used to labour and delivery and because they now have confidence in themselves will prefer to deliver at home”* (Discussant, urban)

One participant was fatalistic in her submission on where a woman is supposed to deliver her baby. She admitted in her words:

*“It is God that has the final say on where a woman will deliver her baby and if she has been destined to deliver at home, no matter how long she stays in the hospital she will still not deliver there until she goes back home”* (Discussant, rural)

The other reasons proffered by the participants on why women deliver at home looked more like excuses. A participant in the urban was of the opinion that home delivery could be incidental in that while the woman may be preparing to go to the hospital labour may start unannounced and she may end up delivering at home. Another participant from the rural area blamed health workers for their poor judgements of labour. She explained that sometimes a woman maybe in active labour but the health worker will tell her to go home and she will eventually deliver at home. Another participant also from the rural area showed a good understanding of the labour process and linked that to delivery at home by the women. She had this to say:

*“Some women have ‘precipitate labour’ and even though they are willing to go to hospital and also have money to pay the hospital bills they may eventually deliver at home because labour started abruptly”* (Discussant, rural)

A participant from the urban area remarked that in some communities in the rural area the health facilities are not easily accessible and based on this some women may deliver at home.

## Discussion

### Decision of where to access antenatal and delivery care

From the results of this study most of the participants in the urban and rural communities were of the opinion that the decision of where a woman should attend antenatal care and obtain delivery services should be a joint decision between the man and the woman. This finding is similar to what was obtained from a quantitative survey in the same study area [21]. A similar finding was obtained from a study in rural communities of Nigeria [22]. Another qualitative study in Ghana revealed that decision making regarding access to and use of skilled maternal healthcare services is strongly influenced by the values and opinions of husbands, mothers-in-law and others more than those of individual childbearing women [23]. In line with the views of some women in the rural communities who were of the opinion that woman should be the ones to decide on where to obtain antenatal and delivery services, an analysis of Bangladesh Demographic and Health Survey revealed that autonomy of women in healthcare decision making was a predictor of use of maternal health service [24].

In the support for joint decisions of husbands and wives on where to attend antenatal care and also deliver, a study in Malawi revealed that decisions taken between husband and wife tend to significantly influence delivery in a health facility when compared to a decision taken independently by either the man or the woman [25]. This brings to the fore the need to involve men in all matters related to maternal healthcare. This need to involve men in the use of maternal health services is further strengthened by the results of a study in north-central Nigeria which revealed that non-permission from husbands was seen as a hindrance to the utilization of antenatal and delivery services [26].

### Role of men in antenatal and delivery services

On the role of men in antenatal and delivery services, all the women sought for intimacy with their husbands during the period of pregnancy. This includes the provision of material and financial needs during this crucial period and to remind and encourage them to attend antenatal care. In a qualitative study involving providers of maternal health services in Ebonyi state southeast Nigeria, all the participants were in agreement that men should be involved in all matters related to maternal healthcare [12]. A community based study that involved couples in Ethiopia established that there is a relationship between male partners’ involvement in maternal healthcare and utilization of some maternal health care services by female partners [27]. For this call to come from the women makes male involvement in issues related to maternal healthcare a necessity. There is also a practical demonstration of the good roles men could play with regards to utilization of maternal health services. For example, a study in rural Nepal revealed that husbands whose wives utilized professional delivery care believed that medical intervention was necessary during delivery and thus provided adequate support. The reverse was the case for women who delivered at home [28]. Thus, there has been a suggestion that health promotion programmes should target the involvement of men in the decision of pregnant women to seek antenatal care so as to encourage adequate use of maternal health services [29].

It could be that the men themselves are aware of their relevance in improving maternal health even though some limitations are associated with this arduous task. For instance, in a study in Morogoro region, Tanzania, men described themselves as supportive of facility based care thus confirming their role as decision makers [30]. However, the men attributed the main barrier to their involvement in maternal health services as lack of knowledge [30]. Perhaps, in a bid to overcome this lack of knowledge a study in Ghana posited that interventions aimed at improving use of maternity services should focus more on different stakeholders at multiple levels especially husbands and mothers-in-law [23].

All the participants in urban and rural communities agreed on their husbands accompanying them during delivery. This is similar to what was obtained from the providers of maternal health care in the study area [12]. However, the women in the rural communities preferred attending antenatal care alone so the money they receive from their husbands will not be decreased should their husbands accompany them. The finding is not entirely new. For instance, the results of a study in rural Nigeria revealed that women are financially dependent on their spouses or partners for pregnancy healthcare related costs. Thus a man’s financial status determined the type of care his spouse or partner sought [22]. The implication of this finding is that a man’s support during pregnancy for his partner was mainly financial [22].

This dependence of women on their husbands or partners for the financial aspects of maternal health care becomes a problem when the man is not financially empowered or less interested in issues related to maternal healthcare. Thus, it has been observed that interventions geared towards supporting women’s financial independence is an important step towards improving their access to skilled healthcare. This was seen as a way to improve women’s decision making capacities [22]. In another clime, it was observed that poverty alleviation strategies will be of relevance in the utilization of antenatal and postnatal care services [31]. Similarly, a study in Gombe state northeast Nigeria revealed that paid work among women was associated with use of health services for delivery services [32]. This necessitates the need for the economic empowerment of women.

In an analysis of Ghana Demographic and Health Survey 2008, the results from the rural population showed that women with some degree of autonomy are more likely to use maternal and child health services than their counterparts who do not have autonomy [33]. In effect, the economic empowerment of women becomes very relevant. Consequently, an analysis of Bangladesh Demographic and Health Survey revealed that the involvement of women in microcredit schemes facilitated the use of maternal health services [24]. Putting an end to the economic limitation of receiving maternal health services prompted the call for outright abolition of financial barriers to antenatal and delivery services [34]. Suffice it to say that countries with high maternal death burden like Nigeria should invest meaningfully in maternal healthcare. To this end, subsidizing maternal healthcare services or abolition of user fees for certain groups of people may be of good effect.

### Choice of where to seek antenatal and delivery services

Most participants in the urban and rural communities were of the opinion that the choice of where to obtain antenatal and delivery services is determined by perceived quality of care. This quality of care is however defined mainly by the experiences of the women or based on the opinion of other women. In a study among providers of maternal health services in urban and rural communities of Ebonyi state, Nigeria, most of the providers attested that the good utilization of their respective health facilities for antenatal and delivery services was due to the good care that they provide [12].

A study in Zambia revealed that trust and quality of care were important when individuals seek facility childbirth [35]. A study in southeast Nigeria concluded that pregnant women look up to the providers of maternal healthcare and their interactions with them and because of this, they are easily satisfied with services received and remain uncritical of the health system [9]. Similarly, a study in Nepal revealed that women could not express what good quality care meant to them. This is because women from low socio-economic status and marginalized ethnicities lack knowledge of their basic reproductive rights thus they judge the quality of care in terms of staff interpersonal behavior and personal experiences [36]. In line with this observation, a study in South Africa emphasized the need to provide women friendly services. This was based on the finding that attitudes of pregnant women about antenatal care are shaped by their knowledge and previous encounters with health services delivery [37].

Thus negative provider-patient interactions including staff inattentiveness and shouting at patients compromised quality of care [38]. In Northern Nigeria, the major reasons for non-utilization of delivery care services included not having a delivery complication and negative provider attitude [39]. In Ghana, a study that involved pregnant women that did not utilize antenatal care, the major health system factor that contributed to the non-utilization of antenatal services was perceived poor attitude of the nurses [40]. In a study that looked at the gap between women’s use of antenatal and delivery services in Northern Uganda, the main barrier to use of delivery services was fear of being neglected or maltreated by health workers. This finding necessitated the call to improve quality of client-provider interaction and respect for women [34]. These findings have necessitated calls for the training of health workers on delivery of quality healthcare [41].

After perceived quality of care, the next factor that determined utilization of antenatal and delivery services was cost. This finding was the same among participants in the urban and rural communities. The issue of cost as a limitation to the utilization of antenatal and delivery services prompted the call for specific interventions for some groups including the urban poor and teenage mothers who are unmarried [21]. This was similar to what was obtained from an analysis of the Demographic and Health Survey of Nepal which concluded that in a bid to improve maternal healthcare, interventions targeting women of low autonomy and in low socio-economic class will be of priority [42]. Suffice it to say that the good involvement of husbands in antenatal and delivery care include bearing the cost involved in that exercise. However, the result of a study in north-central Nigeria revealed that the main barrier to utilization of antenatal and delivery services was lack of money [26]. And in Ghana, the main individual factor that limits the utilization of antenatal care was lack of finance [40].

### Why women deliver at home

Cost appear to be an important factor in the utilization of antenatal and delivery services in Nigeria. From the results of this study, the main reason why the women delivered at home was because of financial constraints. An analysis of the 2008 Nigeria Demographic and Health Survey revealed that money for treatment was the major barrier that prevented women from utilizing maternal health care services [43]. Also, in a study among community members in West Java Province, Indonesia, the major reason why women do not make four or more antenatal care visits or go for postnatal care was financial difficulty including lack of money to pay for health services and transportation [31]. The third factor that influenced choice of health facility was proximity of the health center to the home of the woman. From the results of this, it was seen as a way of defraying transportation cost. However, from the results of another study in the study area, proximity of the health facility to home was the major reason women registered for antenatal care in more than one health facility. This was because the women were aware that events requiring emergency care may occur during pregnancy or at delivery [44].

### Why women patronize traditional birth attendants

Among participants in the rural communities, the rendering of unique services like achieving conception, treatment of malaria and other diseases, cure from contracted pelvis are some of the reasons women patronize traditional birth attendants. These beliefs on the part of the women could be perceived as a form of quality healthcare. In a study in Zimbabwe most traditional medicines were used in the third trimester in a bid to quicken delivery [45]. Women also have other reasons for patronizing traditional birth attendants. In a study in Cross River State, Nigeria, most of the participants still preferred the services of traditional birth attendants despite free maternal health services because they opined that indirect and hidden cost may exist. Thus despite the free maternal health service, they believed that items required for delivery may be out of their reach [46]. In Indonesia, most women preferred to deliver at home or with traditional birth attendants despite the availability of village midwives due to the physical distance and financial constraints. The women implied that facility delivery were meant for women who experienced obstetric complications [47]. Thus it could be said that perceived quality of care, financial constraints and cultural beliefs are some of the reasons women continue to patronize the services of traditional birth attendants.

## Conclusions

All efforts should be made to reduce the huge maternal death burden in Nigeria. This may necessitate the involvement of men and by extension communities in issues related to antenatal and delivery services. There is the need to train health workers in orthodox health facilities on delivery of quality health care. Public enlightenment on the importance of health facility delivery will be of essence. Encouraging women to deliver in health facilities should be prioritized. This may entail the provision of free delivery services or provide subsidized delivery services. The deficiencies of primary health centers especially in the rural areas should be addressed.

## Data Availability

The datasets used and/or analyzed during this study are available from the corresponding author on reasonable request.

## Abbreviations

ANC: Antenatal care
FGD: Focus group discussion
LGA: Local government area
QDA: Qualitative data analysis
TBA: Traditional birth attendant
WHO: World health organization
